# Central Nervous System Metastases in Endometrial Carcinoma: A Case Series

**DOI:** 10.7759/cureus.80464

**Published:** 2025-03-12

**Authors:** Luke Ross, Kelsey Carey, Robert Koenigsberg

**Affiliations:** 1 Radiology, Temple University Hospital, Philadelphia, USA; 2 Neuroradiology, Temple University Hospital, Philadelphia, USA

**Keywords:** bone metastasis, brain metastasis, cns metastasis, endometrial cancer, skull metastasis

## Abstract

Central nervous system metastases from endometrial carcinoma are a rare occurrence and suggest that metastatic endometrial carcinoma can have variable presentations. In this case report, we present three separate cases of patients diagnosed with endometrial carcinoma with metastasis to the central nervous system. The first case presents a woman with grade 2 endometrioid adenocarcinoma with mucinous and clear cell features found later to have skull metastasis with extradural extension. The second case presents a patient with an endometrial yolk sac tumor who, years after her primary diagnosis, was found to have metastasis to the brain. The final case is a woman with endometrial adenocarcinoma who developed brain metastases. While rare, it is important to consider the possibility of the presence of distal bone and brain metastases in patients with endometrial carcinoma. Special attention must be given when these patients report new neurologic symptoms, and one must consider early brain imaging.

## Introduction

Endometrial cancer is the fourth most common cancer in women. About 3% of endometrial cancers occur in women who have an autosomal dominant hereditary predisposition to cancer known as Lynch syndrome. Factors such as early menarche or late menopause, nulliparity, history of diabetes, hypertension, and dietary fat intake have been associated with increased risk for endometrial adenocarcinoma [1].

Both brain and calvarial metastases are an uncommon occurrence in the presence of endometrial cancer. A study performed by Uccella et al. suggests that bone metastases are an uncommon site of disease dissemination in patients with endometrial adenocarcinoma and suggests that this finding occurs in less than 1% of these patients [2]. A case study performed by Mirzaei et al. is one of the few cases that describes the presence of primary bone metastasis to the skull in endometrial adenocarcinoma. Their literature review suggests that there are no previous detailed descriptions of bone metastasis to the skull from endometrial adenocarcinoma without additional osseous involvement. Additionally, skull metastases are generally a late event in the course of disease and often occur after there is already significant disseminated disease. It is rare that skull involvement constitutes the initial site of metastasis [3]. In contrast, brain metastases occur commonly in systemic cancers and account for roughly 40% of intracranial tumors. Primary tumors metastasize to the brain in 9-17% of patients [4]. However, brain metastases due to gynecological malignancies are considered very rare. In a meta-analysis by Karpathiou et al., no more than 3% of brain metastases were attributable to gynecological tumors [5]. Moreover, a study by Piura et al. suggests that 50% of brain metastases from primary endometrial cancer occur in isolation, while the other 50% occur along with systemic metastases [6].

The five-year, age-adjusted survival for endometrial adenocarcinoma has not changed significantly in recent history. The five-year survival rate is the same in 2024 that it was in 1985, 81% [7]. Substantial racial disparities in endometrial adenocarcinoma exist, with an estimated five-year relative survival in 2018 of 84% for white women and 62% for black women. These disparities have been attributed to an increased incidence of tumors with advanced stage, high grade, and aggressive histology in black women [7]. Despite these disparities, the majority of patients who are diagnosed with endometrial adenocarcinoma localized to the uterus have a good prognosis after surgery [1]. However, the patient's prognosis changes significantly with the presence of distant metastases. In these patients, the estimated five-year survival rate is roughly 10%. Additionally, a study conducted by Uccella et al. demonstrated poor survival of endometrial adenocarcinoma patients after diagnosis of bone dissemination with a median survival of one year [2]. The survival rate continues to decline when considering metastases to the brain, with most studies suggesting an average life span of 2.5 to 12.75 months after the diagnosis of brain metastases [8,9].

## Case presentation

Endometrial adenocarcinoma with skull metastasis and extradural extension

A woman in her 70s, with a past medical history of hypertension, arthritis, and uterine fibroids, presented to her gynecologist with six months of intermittent vaginal bleeding. An endometrial biopsy was attempted but was unsuccessful due to the presence of uterine fibroids. The patient subsequently underwent dilation and curettage. This surgery revealed that the uterus was approximately 15 cm, irregularly shaped, with no palpable adnexal masses. The endometrium was felt to be distorted by fibroids. The TrueClear^TM^ device (TruClear Medtronic, Oklahoma City, USA) was used to shave off portions of the uterine fibroid for tissue sampling, and a sharp curettage was done. Pathology showed stage Ib endometrioid adenocarcinoma, grade 2 with mucinous and clear cell features. Immunohistochemical stains showed tumor cells positive for vimentin, ER, and PR, while being negative for carcinoembryonic antigen (CEA) and Napsin-A. P16 showed patchy strong positivity and a p53 stain showed wild-type expression. Due to these findings, the patient underwent a total abdominal hysterectomy, a bilateral salpingo-oophorectomy, and bilateral pelvic lymphadenectomy. Due to her high-risk features, the depth of invasion, the stage of the tumor, and her being over 70 years old, the patient was treated with vaginal brachytherapy (30 Gy in 5 fractions). She did not receive chemotherapy at this time.

**Figure 1:**
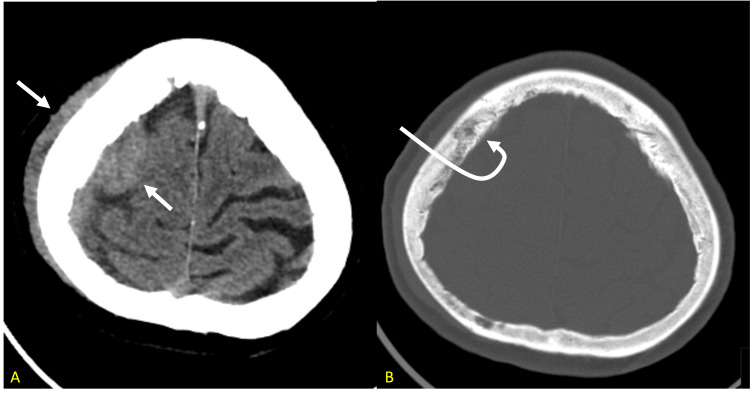
a. CT head (soft tissue window) demonstrating a right frontal extra-axial lesion and an extracranial soft tissue component within the scalp. b. The bone window suggests subtle permeative bone marrow infiltration of the right parietal bone.

Twelve months later, the patient presented to the emergency department with left upper extremity weakness and abdominal pain. The patient reported that she was unable to move her left arm or hold anything in her left hand. She reported that her hand also began to feel numb. She also noted that she had a lump on the right side of her head that had been increasing in size over the past month. She denied any head trauma, headache, lightheadedness, dysarthria, dysphagia, or syncope. CT chest, abdomen, and pelvis were negative for metastatic disease. A CT head and MRI brain were then performed. The CT head demonstrated a right parietal extra-axial lesion with adjacent lytic changes within the calvarium and a soft tissue component within the scalp (Figures 1a, 1b).

**Figure 2:**
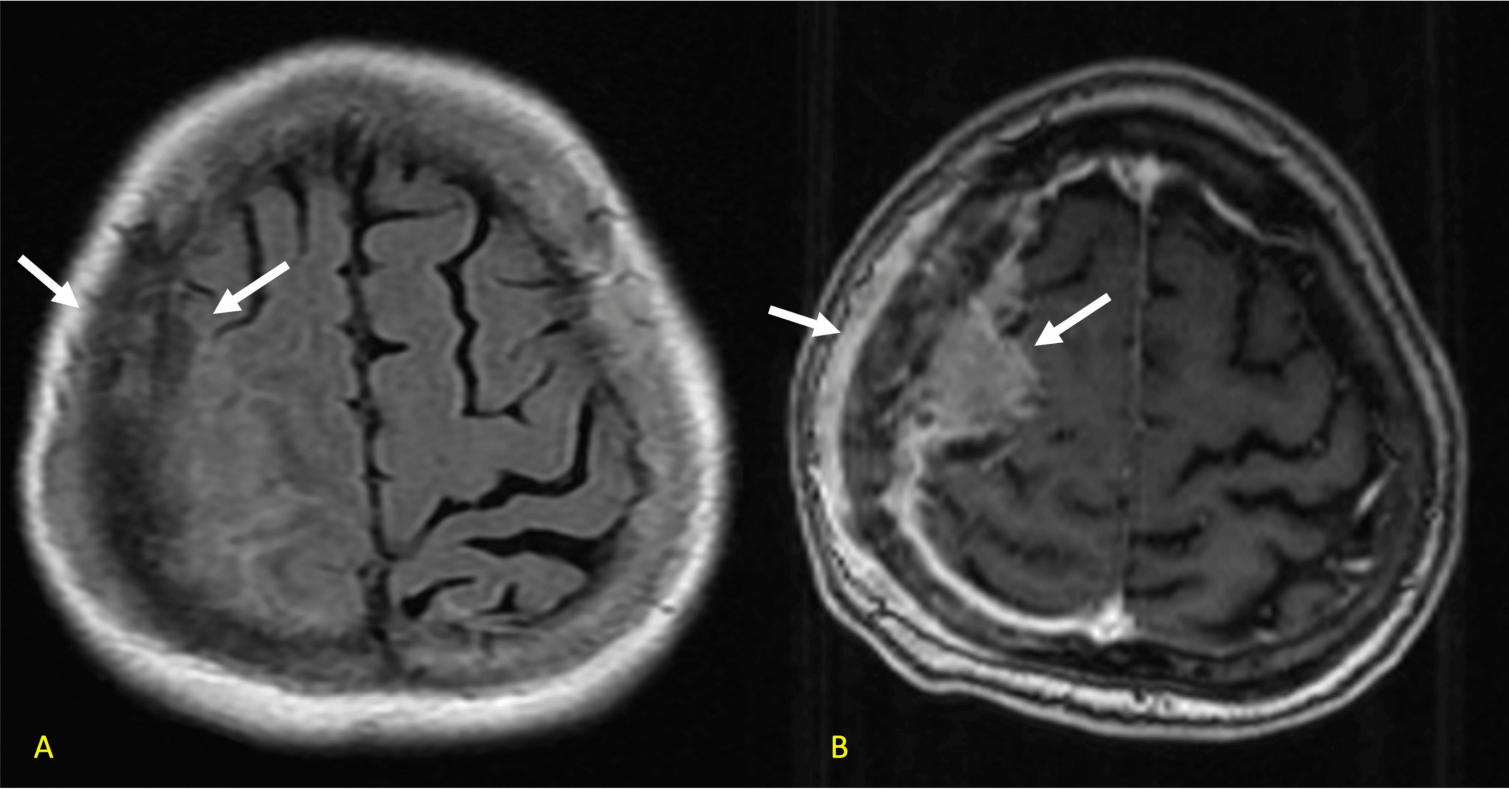
a. Axial T1 MRI brain images demonstrating metastatic infiltration of the right parietal bone demonstrating low signal marrow compared to the normal marrow fat high signal seen on the left. b. Contrast axial T1 showing mild diploic space enhancement with enhancing subgaleal and epidural masses along the right fronto-parietal convexity with adjacent frontal lobe compression.

Differentials included a large transcalvarial meningioma, metastatic lesions, and a subdural hemorrhage with an overlying scalp hematoma. MRI brain was subsequently performed and showed a large right frontoparietal temporal convexity lesion, with intraosseous and extracranial extension of the lesion (Figures 2a, 2b).

**Figure 3:**
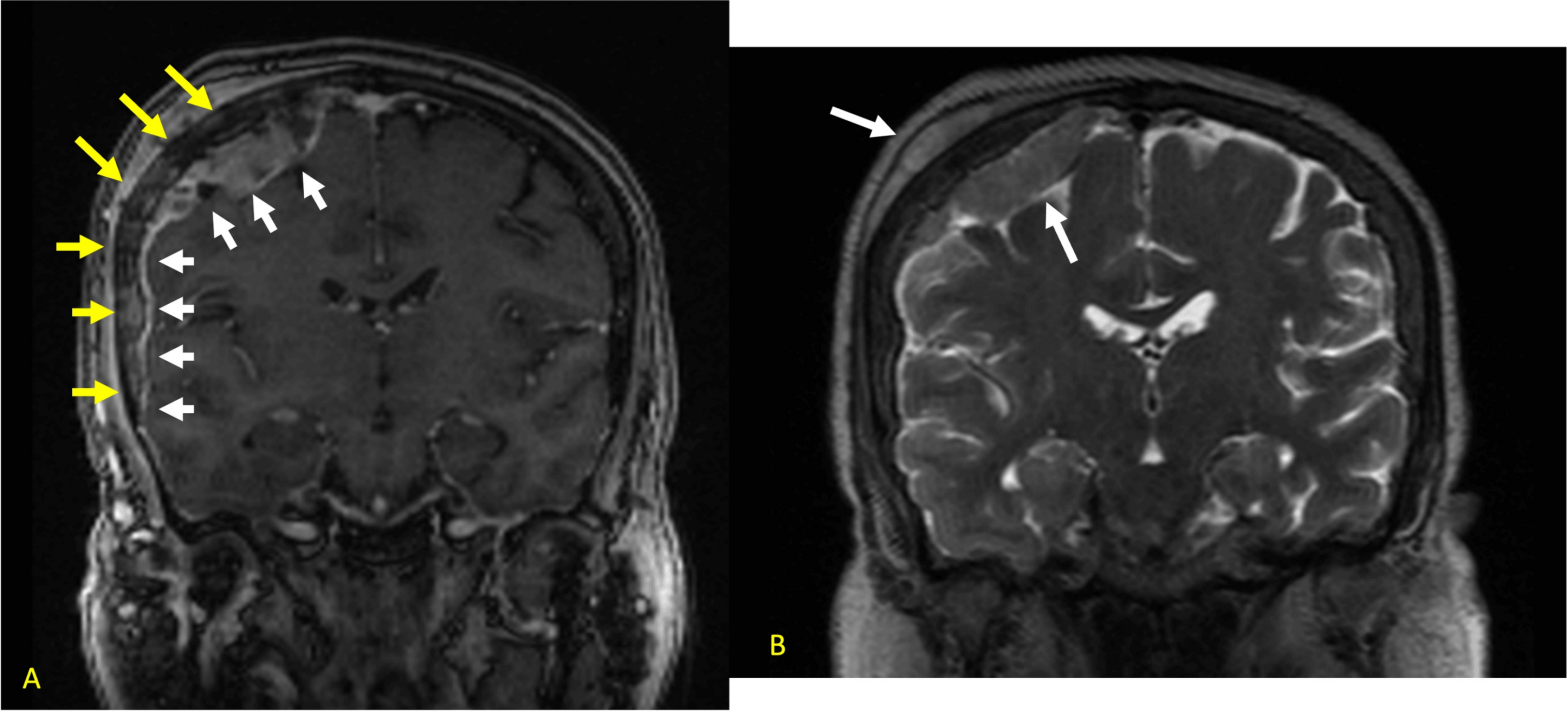
a. Coronal T1 MRI brain image demonstrating epidural disease with a long segment of dural infiltration, skull invasion, and a large extracranial component elevating the skin. b. Coronal T2 MRI demonstrating brain displacement without cerebral edema.

There was additional diffuse dural enhancement throughout the right cerebral convexity. Along the thickest portion of right frontoparietal extra-axial enhancing tissue, the region measured approximately 2.4 x 2.0 x 1.3 cm (Figures 3a, 3b).

The patient then underwent a right parietotemporal craniotomy. The postoperative CT head demonstrated that the right parietal extra-axial mass had been surgically removed with extra-axial air along the right frontal parietal convexity. The pathology collected from the craniotomy demonstrated adenocarcinoma with mucinous features, compatible with metastasis from the patient's primary endometrial cancer. A subsequent MRI brain was performed one month later, demonstrating worsening right parietal dural thickening. There were also persistent abnormal bone marrow changes involving the right frontal and parietal bone margins, with persistent extracranial subgaleal metastatic extension. The patient began whole brain radiation therapy (30Gy in 10 fractions) and was put on letrozole (2.5mg daily).

Endometrial yolk sac tumor with brain metastasis

A woman in her 70s with a past medical history of diffuse large B-cell lymphoma status post five cycles of EPOCH (etoposide, prednisone, vincristine, cyclophosphamide, doxorubicin, and rituximab) presented to the emergency department with vaginal bleeding. Her workup at the time included a pelvic ultrasound, which showed diffuse endometrial thickening. She then underwent a dilation and curettage hysteroscopy, after which she underwent endometrial curettage that showed rare, atypical glandular epithelium and necrotic debris, favoring a neoplastic process. Immunohistochemical stains demonstrated the atypical glandular epithelium to be positive for mCRA and p16 while negative for ER and vimentin, making it worrisome for endocervical origin. After the pathology diagnosis, she underwent a CT abdomen and pelvis that showed thickening of the endometrium concerning for malignancy and no metastatic disease. She subsequently underwent a total abdominal hysterectomy with bilateral salpingo-oophorectomy and bilateral pelvic lymph node dissection. Pathology was reviewed, and she received the diagnosis of FIGO stage IA pT1a pN0 cM0 yolk sac tumor of the endometrium. She was then treated with four cycles of cisplatin (20mg/m²) and etoposide (75mg/m²) therapy, along with radiation therapy. A year later, a screening CT of the chest revealed a right upper lobe lesion, which biopsy confirmed as a metastasis from her primary endometrial cancer. For her metastatic disease, she was initiated on carboplatin (AUC 5) and paclitaxel (175mg/m²) in addition to radiation therapy. Subsequent chest CT scans three months later revealed an increasing number of pulmonary lesions suspicious for metastasis. Her chemotherapy regimen was then switched to pembrolizumab (200mg every three weeks) and lenvatinib (4mg daily), with follow-up CT scans showing relatively stable disease.

**Figure 4:**
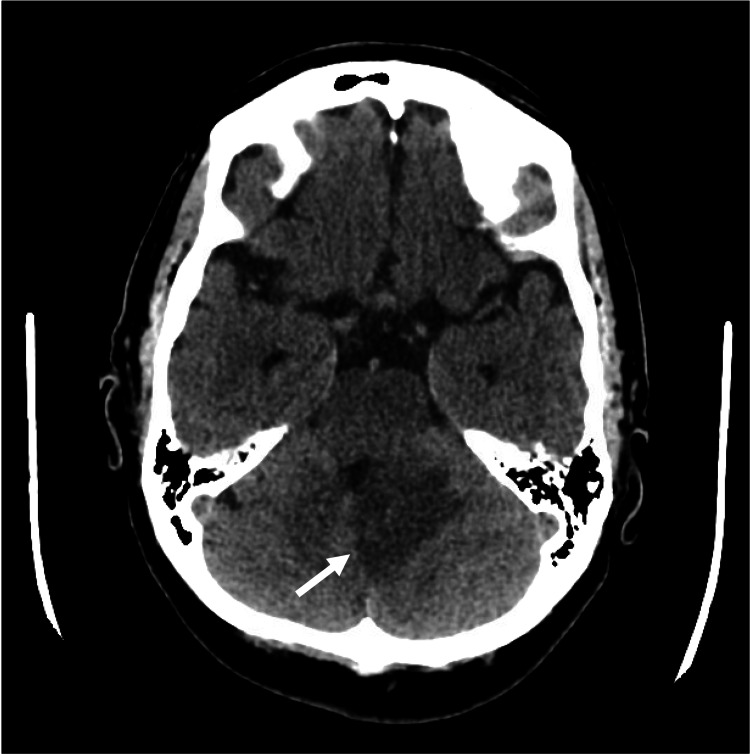
CT head demonstrating left cerebellar vasogenic edema with mass effect on the fourth ventricle.

A year later, she presented to the emergency department with recurrent nausea and emesis. A CT scan of the head showed a large, left cerebellar lesion with surrounding edema and mass effect (Figure 4).

**Figure 5:**
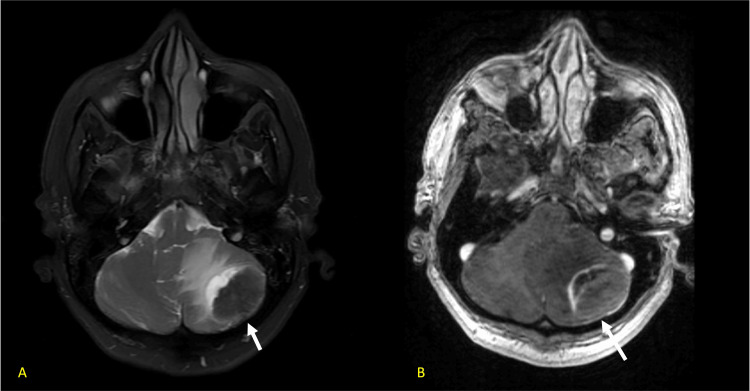
a. T2-weighted axial image through the posterior fossa demonstrating a low signal peripheral mass with adjacent vasogenic edema. b. Post-contrast T1 imaging redemonstrating mass with soft tissue and capsular enhancement.

To further characterize the lesions, she received an MRI brain with and without contrast that showed a well-circumscribed mass in the left cerebellum measuring 3.4 x 2.9 x 2.7 with the imaging characteristic concerning for metastatic disease (Figures 5a, 5b).

**Figure 6:**
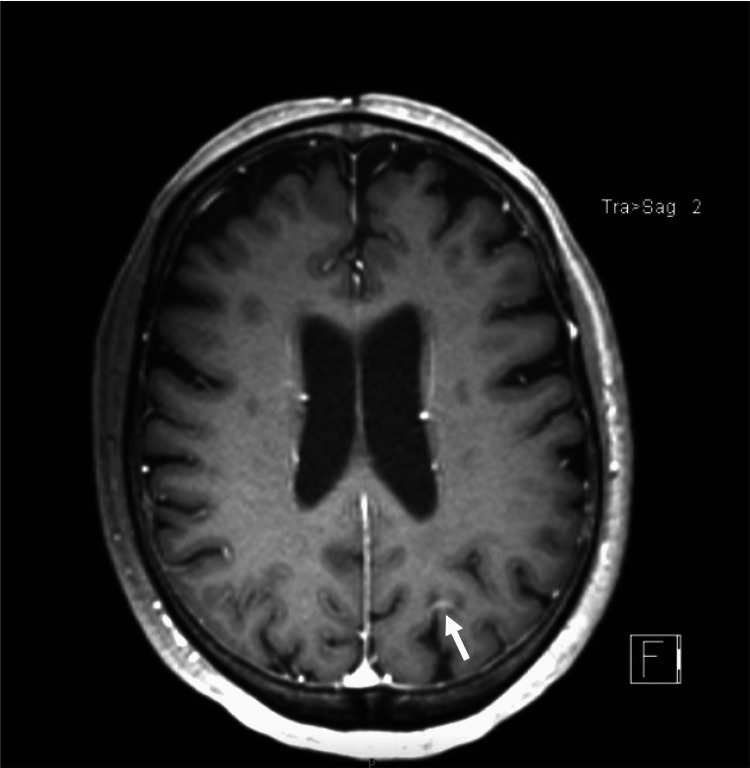
Post-contrast T1 image showing new linear left parietal enhancement representing new metastatic focus.

She underwent neoadjuvant Gamma Knife irradiation (1600 cGy in 1 fraction) of the mass and subsequent suboccipital craniotomy for resection of the tumor. Pathology showed glandular formation and extensive necrosis compatible with her endometrial yolk sac tumor. Four months later, a repeat brain MRI demonstrated focal areas of enhancement within the deep inferior to cerebellar parenchyma resection margins, and along the superiorly and anteriorly, globular dural-based enhancement suspicious of tumor recurrence (Figure 6).

For this, she underwent stereotactic radiosurgery. Three months later, follow-up scans showed increasing pulmonary lymphadenopathy that, following diagnostic bronchoscopy, were positive for recurrent endometrial metastasis. The pathology showed that they were positive for HER2, and she was started on trastuzumab deruxtecan, which she is still currently taking. Follow-up scans of her head have shown control of her intracranial disease.

Endometrial adenocarcinoma with brain metastases

A woman in her 40s presented to the emergency department for constipation. She was noted to have an 11 cm cyst on CT at this visit. Later the same year, she developed back and abdominal pain. Repeat imaging showed a large complex pelvic mass measuring 13.8 cm. Diagnostic laparoscopy was performed along with total abdominal hysterectomy and bilateral salpingo-oopherectomy. At the time, intraoperative findings were remarkable for a 15cm left adnexal mass with carcinomatosis noted in the pelvis and within the omentum. Her pathology showed high-grade serous carcinoma involving her left ovary and fallopian tube, omentum, peritoneum, right ovary, and bilateral pelvic lymph nodes. Pathology results were reviewed, and she was diagnosed with stage IVA high-grade serous endometrial cancer. Differential diagnosis included synchronous stage IA high-grade serous endometrial cancer and stage IIIA2 high-grade serous ovarian cancer; however, molecular profiling of both the endometrial and fallopian tube cancers revealed one etiology. After an interdisciplinary meeting, it was highly favored to be primary endometrial cancer.

She was started on adjuvant carboplatin (AUC 6) and paclitaxel (175mg/m^2 ^every three weeks). Follow-up CT scans showed persistent disease, and she began pembrolizumab (200mg every three weeks) and lenvatinib (20mg daily).

**Figure 7:**
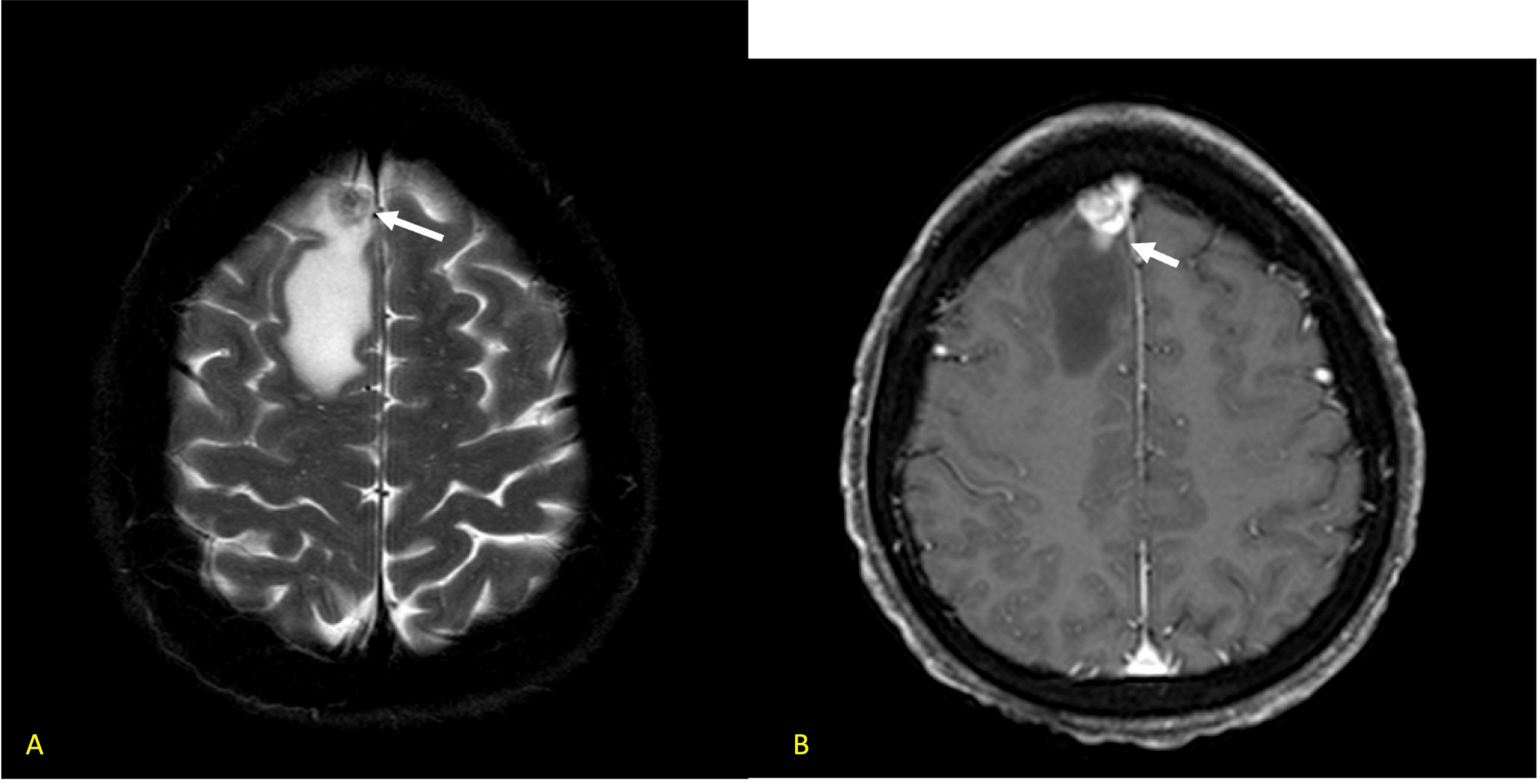
a. Axial T2 image demonstrating low signal right frontal mass with adjacent vasogenic edema. b. Image showing the mass heterogeneously enhancing post-contrast.

After numerous cycles of treatment, her disease remained stable; however, two years later, she reported daily headaches and new vertigo, for which she was referred to neurology. She underwent brain MRI which showed a 1.6 cm enhancing mass in the right frontal lobe with associated vasogenic edema (Figures 7a, 7b).

She was placed on dexamethasone (6mg q8hr, tapered as symptoms improved), leading to significant symptom relief. She then underwent a right frontal craniotomy for tumor resection. Pathological analysis confirmed high-grade serous carcinoma from a primary Müllerian tumor, consistent with her known endometrial disease. She subsequently underwent adjuvant brain stereotactic radiosurgery (27 Gy in three fractions). Given the progression of her disease on pembrolizumab (200mg every three weeks) and lenvatinib (20mg daily), she was switched to carboplatin (AUC 5) and paclitaxel (175mg/m² every three weeks).

**Figure 8:**
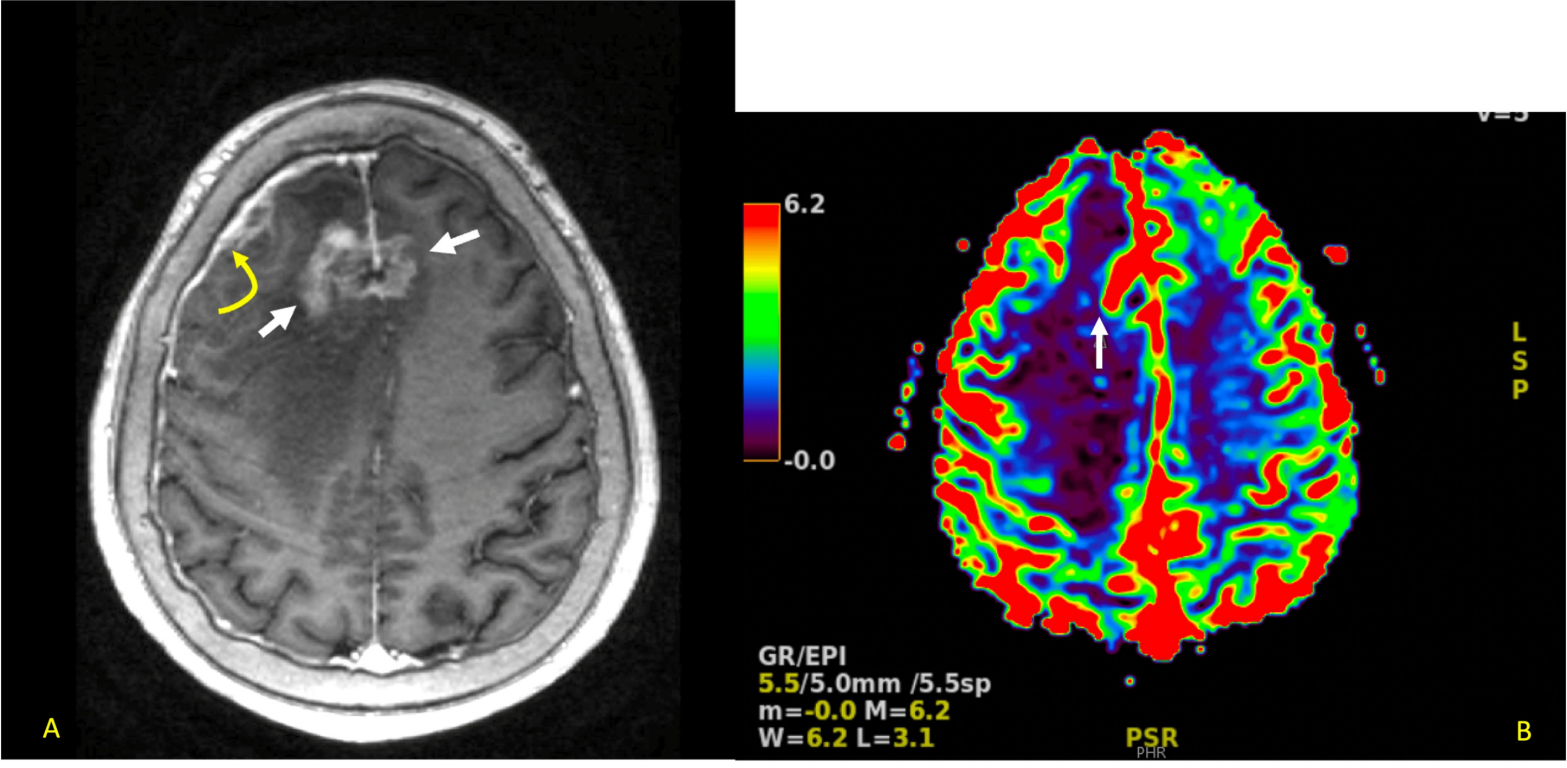
a. Extensive bifrontal recurrence surrounding the falx cerebri favoring tumor rather than radiation necrosis. Arrows showing involvement of right and left hemispheres and the curved arrow showing extensive right dural enhancement, likely metastatic infiltration. b. Cerebral blood flow perfusion image demonstrating increased perfusion, highly suggestive of a tumor rather than radiation necrosis.

Her first follow-up MRI two months after surgery demonstrated no new metastatic lesions as well as resolution of the vasogenic edema. Subsequent imaging seven months after surgery revealed an increased size of the mediastinal and retroperitoneal lymph nodes consistent with metastatic disease. MRI brain three months later showed the development of several new cortical metastatic lesions causing vasogenic edema and regional mass effect with a midline shift (Figures 8a, 8b).

Given the high number of lesions and their proximity to the prior surgical cavity, surgery was deferred, and she underwent whole brain radiation therapy (30 Gy in 3 fractions) as well as doxorubicin therapy (40mg/m² infusion every 21 days). As of this time, she remains on the same regimen. 

## Discussion

Central nervous system metastases from endometrial adenocarcinoma are a rare occurrence and suggest that metastatic endometrial adenocarcinoma can have variable presentations [5]. While extremely rare, it is important to consider the possibility of the presence of distal bone and brain metastases in patients with endometrial adenocarcinoma. Early diagnosis and treatment of these metastases can improve patient survival and are important considerations when evaluating patients with endometrial adenocarcinoma [5].

Brain imaging is a critical tool in the diagnosis of central nervous system metastasis. CT scans with bone windows are a useful tool to identify lytic bone lesions. Radiographs have limited utility in the diagnosis of calvarial meningiomas because of the superimposed bony structures; however, CT with bone windows is necessary in the detection of the tumor, cortical destruction, and both intra- and extraosseous extension [10]. A significant limitation of CT is that it does not clearly show the various degrees of dural invasion and has comparatively poorer spatial resolution as compared to MRI. MRI with and without contrast is superior to CT for skull metastasis detection. The primary finding in skull metastases consists of substitution of the usual hyperintense fat sign by a hypointense lesion on T1-weighted images with a variable appearance on T2-weighted images. MRI is especially useful in demonstrating any invasion into the dura or cranial nerves [11]. Imaging is also a critical component in the diagnosis of brain metastasis. A study by Chura et al. showed that the diagnosis of brain metastases was established with both CT and MRI in 40% of patients, MRI alone in 40% of patients, and CT alone in 20% [12]. Gadolinium-enhanced MRI has higher sensitivity (77% vs. 21%) than positron emission tomography combined with computed tomography (PET/CT). However, PET/CT does have a high specificity [13]. In the cases described, the lesions were identified on CT and/or MRI, and thus, PET/CT was not necessary.

The treatment for both brain and skull metastasis includes radiotherapy, chemotherapy, and surgery. The current standard of treatment includes either radiotherapy alone or radiotherapy in conjunction with chemotherapy and surgery. In calvarial metastasis, studies suggest that radiotherapy provides significant pain reduction, as well as improvements in cranial nerve function. Additionally, the speed with which radiotherapy is initiated plays a significant role in patient outcome. If the radiotherapy is started less than one month after the onset of symptoms, patients typically have an increased rate of improvement in focal neurologic deficits. A study conducted by Vikram and Chu found that 87% of patients with calvarial metastases improved after radiotherapy if symptoms were present for less than one month, as opposed to only 25% if symptoms were present for three months or more [14]. 

One possible treatment option for patients with isolated (limited to the brain only) and single (solitary) brain metastases is resection of the brain metastases by craniotomy, followed by whole brain radiotherapy (WBRT). While this method is still undergoing clinical trials, it is becoming a more widely accepted treatment option. Patients with multiple brain metastases generally would receive WBRT alone. Research does suggest that multimodal therapy (both craniotomy and WBRT) is more beneficial than a single approach [9]. While not yet well documented, stereotactic radiosurgery will likely become a more popular treatment option as it limits the significant neurotoxicity that can be seen with whole-brain radiotherapy [15].

## Conclusions

In the presence of endometrial adenocarcinoma, brain and skull metastases occur very rarely. These metastases can not only cause pain but can also result in localized expansion, which can cause serious neurologic deficits. While rare, it is important to consider the possibility of central nervous system metastases in patients with high-grade endometrial adenocarcinoma. Early and continuous metastatic screening, including brain imaging, as well as early evaluation by the oncology team, may enable early detection and improve patient outcomes.
